# Gut Microbiota: A Potential Role in Modulating Carcinogenesis and Response to Anti‐Cancer Therapies

**DOI:** 10.1002/cnr2.70585

**Published:** 2026-05-21

**Authors:** Awgichew Shewasinad Yehualashet, Eleni Teklu Fersha, Berhan Begashaw Yikna, Kassahun Dires Ayenew

**Affiliations:** ^1^ Department of Pharmacy College of Health Sciences, Debre Berhan University Debre Berhan Ethiopia

## Abstract

**Background:**

The effects of microbiota on cancer are not only limited to their role in oncogenesis, progression and response to anticancer therapies; the disease itself is also influenced by microbiota. In recent investigations, alterations in the gut bacterial community between cancer patients and healthy individuals have been reported. Outcomes targeted to gut microbiota composition, microbial metabolites, immune responses, and other pertinent measures are also aggregated and elaborated in this review.

**Recent Findings:**

Numerous preclinical and clinical experiments have explored the use of gut microbiota as a new research avenue with encouraging outcomes in enhancing treatment efficacy and mitigating side effects related to treatment. The pathophysiology of cancer cells and modulation by microbiomes via many signaling pathways could be useful in determining the role of microbiota in developing novel therapeutic approaches and possible targets for cancer treatment.

**Conclusion:**

Though different bacterial species can cause various clinical outcomes, more research is still required to identify more precise and profound underlying mechanisms in the relationship between gut microbiota and anticancer therapy. This could lead to improved patient outcomes overall and more focused clinical applications down the road.

## 1 Introduction

### 1.1 Background

The human microbiota is made up of more than 100 trillion different species, including fungus, viruses, bacteria, and protozoa. The human microbiome is mostly influenced by the human gut as compared to other parts of the human body [1]. Although, it is commonly believed that a newborn's gut is sterile, it is promptly colonized after delivery. The delivery techniques, early childhood feeding (i.e., formula vs. breast‐feeding), host genetics, and a variety of environmental exposures, such as diet and dietary habits, all affect the composition and diversity of microbiota [2]. The various gastrointestinal tract areas exhibit significant variations in transit time, PH, oxygen exposure, nutrition availability, host secretions including bile and digestive enzymes, mucosal surfaces, and immune system interactions. These factors collectively impact the process of microbial colonization [3].

The microbiome, which is 150 times larger than the human genome, is the aggregate name for the genes of the microbiota. The majority of bacteria in an individual are 150–170 species, which benefit from the warm, nutrient‐rich environment of the gut and carry out protective, metabolic, and structural tasks [2]. The human body benefits from the gut microbiota's critical role in the synthesis of vitamin B and K, the generation of short chain fatty acids (SCFAs) from dietary fiber, the metabolism of various substances, including xenobiotics and sterols, and the control of immunological responses. Furthermore, through a variety of physiological processes, including preserving gut integrity or forming the intestinal epithelium, absorbing energy, warding off infections, and controlling host immunity, the microbiota provides numerous advantages to the host. However, dysbiosis, or a changed microbial makeup, has the potential to upset these systems [1, 3].

Microbial dysbiosis may contribute to the onset of several disorders including cancer because of modifications to the metabolism of microorganisms (Mos), which alter the release of metabolites such as bile acids (FBAs), phenolic compounds, and SCFAs or bacterial components that affect the host's gut essential activities [4].

### 1.2 Bacterial Metabolites Associated With Oncogenesis

Although microbiota in the various parts of our body can synthesize metabolites, it is the intestinal microbiota which is unique in terms of metabolite production as the intestinal microbiota represents the largest density and abundance of microbiome in the human body. Intestinal microbiota and its metabolites can also travel to other parts of the body via the bloodstream and influence inflammation and the development of tumors in individual organs employing the gut–brain axis, gut–lung axis, and gut–hepatic axis [5].

With the process of digestion, the gut microbiome produces different metabolites including SCFAs, trimethylamine N‐oxide (TMAO), microbial tryptophan catabolites (MTCs), polyamines (PAs), hydrogen sulfide and secondary BAs, protein‐bound uremic toxins (PBUTs), branched‐chain amino acids (BCAAs), and various uncharacterized molecules. These metabolites play crucial roles in host–microbe interaction, metabolism, immune modulation, and physiological homeostasis [6].

SCFAs are the primary metabolites produced by the intestinal microbiota through the metabolism of undigested carbohydrate, protein, and fat. As compared to other microbial metabolites, SCFAs, MTCs, PAs, hydrogen sulfide, and secondary BAs have significant impacts on the development of tumors [5].

Various Studies on microbial metabolites in human blood and urine have revealed the strong correlation between the production of microbial metabolites and human tumors. If the production of metabolites is abnormal, it can potentially impact tumor formation and progression as shown in Table 1 below. Investigations also demonstrated their involvement in the occurrence and development of tumors [5].

**TABLE 1:** Microbial metabolites in human blood and urine and their correlation with tumor development and progression.

Cancer type	Sample type	Identified metabolite	Possible mechanism	References
Multiple types of cancer	Blood	SCFAs, tryptophan metabolites (indoles), BAs	Microbial metabolome influences drug response and toxicity	[7]
	Blood/tissues	SCFAs (butyrate), secondary BAs, PAs	Regulate tumor cell function and response to immunotherapy	[8]
	Blood/systemic	SCFAs, inosine, BAs	Regulate immune responses in (TME) tumor microenvironment	[9]
	Blood/tissues	SCFAs (butyrate), histone modifying metabolites	Influence tumor epigenetic regulation	[9]
Bladder cancer	Urine	Aromatic amines, indoles, phenolic compounds	Contribute to carcinogenesis and inflammation	[10]
	Urine	Nitrosamines, aromatic compounds, microbial toxins	Leads to DNA damage and inflammation	[11]
	Urine	Nucleosides, amino acid metabolites, microbial degradation products	Altered with tumor presence and removal	[12]
Colorectal cancer	Blood/urine/stool	SCFAs (acetate, propionate, butyrate), secondary BAs	Non‐invasive biomarkers; influence tumor progression	[13]
	Blood	SCFAs, BAs, BAs	Influence prognosis and disease progression	[14]
Liver (Hepatocellular carcinoma)	Blood/serum	BAs, amino acid derivatives, lipopolysaccharide‐related metabolites	Strong microbiota–metabolite correlation in cancer patients	[15]
Pancreatic cancer	Blood	Branched‐chain amino acids, lipid metabolites, microbial co‐metabolites	Associated with increased cancer risk	[16]
Breast cancer	Blood	Lipid metabolites, estrogen‐like microbial metabolites, SCFAs	Distinguish cancer patients from controls	[17]
Lung cancer	Blood/serum	Lipid metabolites, amino acid derivatives, oxidative metabolites	Correlate with microbiome alterations	[18]

Gut microbial metabolism plays a central role in CRC development as dysbiosis converts dietary components into harmful metabolites, such as secondary BAs and hydrogen sulfide, which induce oxidative stress and DNA damage in colonic epithelial cells. The metabolites further exacerbate the tumor‐promoting effects of microbiota as a result of dysbiosis [19]. Therapeutically, the gut microbiota can be targeted to potentially prevent and treat CRC. Probiotic and prebiotic interventions can restore microbial balance and enhance antitumor immunity. Fecal Microbiota Transplantation (FMT) has also been explored to counteract dysbiosis in CRC patients [20].

From the view of gut microbiota, CRC is one of the most extensively studied cancer types; associating dysbiosis onset and progression of the disease. *F. nucleatum* is highly abundant in CRC tissues and is known to promote carcinogenesis with different mechanisms as it adheres to colonic epithelial cells by its adhesin protein FadA, that binds to E‐cadherin, activating β‐catenin pathways that provoke cell proliferation and tumor invasion. *F. nucleatum* also enhances the immunosuppressive TME using MDSCs while inhibiting the activity of cytotoxic T cells, enabling immune evasion by tumor cells [21].

*F. nucleatum* is broadly associated with pro‐tumorigenic immune modulation; however, conflicting findings suggest that its abundance varies by tumor stage, functional site, and host immune tone. Similarly, metabolites such as deoxycholic acid and succinate can exhibit both genotoxic and signaling‐regulatory roles, depending on concentration and host context. Protective SCFAs like butyrate may shift toward pro‐oncogenic activity under hypoxic or dysbiosis. Moreover, while the Wnt/β‐catenin pathway is often activated by microbiota‐derived signals, recent studies suggest microbial influence may be secondary to tumor‐intrinsic signaling dysregulation [22].

*E. coli* is another key regulator in CRC, specifically strains harboring the polyketide synthase genomic island which produces colibactin, resulting in genomic instability and the accumulation of mutations in colorectal tissues. Dysbiosis creates an inflammatory environment that further exacerbates genotoxicity [21]. Furthermore, *B. fragilis* , particularly its enterotoxigenic strains, is also a player in CRC by secreting BFT, which is a toxin that interrupts epithelial barrier integrity and induces chronic inflammation. BFT‐mediated activation of the Wnt/β‐catenin pathway is a potential mechanism that promotes epithelial cell proliferation and enhances carcinogenesis [21, 23].

### 1.3 Functional Characteristics of Microbial Metabolites in Tumors

SCFAs, produced by microbial fermentation of dietary fiber, serve as key metabolic intermediaries linking microbiota to host epigenetic regulation. Butyrate, acetate, and propionate act as signaling molecules by inhibiting class I and III histone deacetylases (HDACs) and activating G protein–coupled receptors (GPR41 and GPR43), thereby promoting interleukin‐22 (IL‐22) secretion and maintaining mucosal homeostasis. Conversely, butyrate, a classical HDAC inhibitor, also suppresses the Wnt/β‐catenin signaling pathway by disrupting β‐catenin complexes and restoring differentiation markers in tumors. Microbial dysbiosis or reduced butyrate availability impairs this regulatory axis and promotes oncogenic activation, whereas supplementation of exogenous butyrate may help reverse tumor stemness. In addition, HDAC inhibition, SCFAs regulate cytokine production, neutrophil migration, and the generation of neutrophil extracellular traps (NETs), thereby alleviating inflammation and preserving epithelial barrier integrity. This integrates metabolic, immune, and epigenetic regulation to maintain intestinal and oncologic homeostasis [24].

Microbial metabolites are not only metabolic substrates or cofactors but they can also actively modulate tumor signal transduction, metabolic reprogramming, and the immune microenvironment via various molecular mechanisms. For example, secondary BAs and SCFAs regulate tumor signaling via the Wnt/β‐catenin and PI3K/AKT/mTOR pathways. Lactate regulates cellular energy metabolism through monocarboxylate transporters (MCTs), including MCT2 and MCT4. Indole‐derived and related microbial metabolites can induce oxidative and endoplasmic reticulum stress. Moreover, additional microbiota‐derived metabolites influence the fate of tumor cells by remodeling the immune microenvironment and modulating immune regulatory networks [25].

SCFAs can also remodel the TME through promoting tumor progression and metastasis with immunomodulatory effects, regulating signal transduction and modulating inflammation. Besides, SCFAs enhance activation of the MAPK and PI3K signaling pathways in the neighboring prostate region by upregulating the production of insulin‐like growth factor‐1, thereby facilitating the proliferation and multiplication of prostate cancer cells [24]. Gut microbiota derived bile salt hydrolases (BSHs) convert primary BAs into secondary BAs, mainly deoxycholic acid (DCA) and lithocholic acid (LCA). Recent studies revealed that increased levels of these microbial metabolites are linked with the progression of CRC by enhancing epithelial proliferation, inflammation, and DNA damage [26]. Secondary BAs also enhance tumorigenesis by activating oncogenic signaling pathways, including Wnt/β‐catenin, which contributes to tumor growth and malignant progression [27]. In addition, microbiota‐driven BA metabolism modulates the tumor immune microenvironment by promoting Treg infiltration and suppressing cytotoxic T cell activity via chemokine and receptor mediated signaling, hence facilitating immune evasion in CRC [26, 27].

Additionally, secondary BAs also induce the expression of cytokines and affect tumor progression. LCA activates Erk1/2 MAPK and inhibits STAT3 phosphorylation to promote IL‐8 expression in colorectal cancer cells. LCA induces the progression and metastasis of CRC via promoting IL‐8 expression. Furthermore, DCA activates CD4^+^ T cells and induces IL‐10 secretion to induce Treg cell differentiation, causing an immunosuppressive microenvironment. Furthermore, the release of IL‐10 stimulates the polarization of tumor‐associated macrophages (TAMs) towards the M2 phenotype, hence facilitating the proliferation and metastasis of CRC. The two derivatives of LCA are 3‐oxo LCA and isoallo LCA. 3‐oxo LCA hinders Th17 cell differentiation by binding to retinoid‐related orphan receptor‐γt, whereas isoallo LCA encourages the formation of Tregs by producing mitochondrial reactive oxygen species (ROS) [24].

### 1.4 Contemporary Treatment Landscape for Anti‐Cancer Therapy

Next to cardiovascular illnesses, cancer is the second most common cause of early death worldwide, accounting for one out of every six fatalities [28]. The random accumulation of spontaneous mutations that take place inside cells during deoxyribonucleic acid (DNA) replication is the origin of cancer. Furthermore, environmental exposure and lifestyle decisions can have a big impact on a person's chance of getting cancer [29]. In 2020, 19.3 million new instances of cancer were reported, and about 10.0 million of those cases resulted in cancer‐related deaths. There will be 28.4 million instances of cancer worldwide in 2040 [30].

Although substantial progress has been in place in both early detection and therapeutic innovation, conventional treatment modalities, surgery, radiotherapy, and chemotherapy are still fraught with shortcomings with their systemic toxicity, tumor recurrence, and therapeutic outcome [31]. The major concerns in the use of phytochemical substances to treat cancer include their poor water solubility, inability to penetrate targeted cells, absorption by normal cells, limited therapeutic potential, and harmful side effects [32, 33]. Currently, many drugs have received formal worldwide approval for the treatment of cancer; however, cancer‐related deaths remain incurable [34]. As the therapeutic index ratio for all chemotherapy drugs is low, an increased dose is likely to result in significant morbidity, mortality, or both in the absence of other interventions, such as stem‐cell transplantation [35, 36].

In recent decades, breakthroughs in molecular biology, immunology, and biotechnology have driven the development of more precise and personalized cancer treatments. These include targeted therapies, monoclonal antibodies, antibody–drug conjugates, immunotherapies, cell and gene therapies, epigenetic modulators, metabolic interventions, and nanomedicine. Novel computational approaches, such as Boolean network modeling, are now being applied to identify key regulatory targets for cancer reversion. Despite the promise of these emerging modalities, significant challenges remain, including high costs, treatment accessibility, immune evasion mechanisms, and diagnostic limitations [31].

## 2 Microbiota and Role in Carcinogenesis

Changes in the composition of the gut microbiota have been linked to the onset and progression of cancer in several different tissues, including melanoma, gastric cancer, CRC, hepatocellular carcinoma (HCC), pancreatic cancer, and breast cancer. Alterations in the bacterial community of the gut between cancer patients and healthy individuals have been reported in recent investigations [37].

To maintain homeostasis, the host immunity controls the gut microbiome, and the microbiota in turn influences host immunity. The immune system in distal mucosal locations can be influenced by the gut microbiome through immunological regulation, systemic metabolism, and circulation, in addition to gut immunity. There are three stages in the interaction between immunity and tumor cells. These phases are: tumor immune clearance, immunological balance, and immune escape. Immunological equilibrium is the dynamic balance by which cancer cells cannot be entirely eradicated or develop rapidly because they are still controlled by immunity. Immune elimination refers to the recognition and removal of tumor cells by immune cells. The final stage, known as immunological escape, sees released tumor cells proliferate apart from the host immune system. Consequently, immunity has a dual role in tumorigenesis [38].

The gut microbiome can cause cancer through a variety of mechanisms, including the direct oncogenic effect of MOs and their products, changes in circulating metabolites that become pro‐carcinogenic, inducing pro‐inflammatory and immunosuppressive pathways to disrupt host cancer immunosurveillance, and stimulating the synthesis of trophic factors by the host as presented in Table 2 [52]. In addition, a number of variables, including the host's lifestyle, the consumption of antibiotics, and the microbiome transplantation itself, influence the development of cancer and reaction to cancer treatment [1].

**TABLE 2:** Gut microbiota and association with carcinogenesis.

Microbiota and mechanism of carcinogenesis in colorectal cancer (CRC)		
Bacterial species	Mechanism of carcinogenesis	References
*Fusobacterium nucleatum*	Provoking NF‐κβ signaling cascade to promote Wnt signaling pathway	[39]
*Escherichia coli* NC101	Production of genotoxins	[40]
*Streptococcus gallolyticus*	IL‐1, IL‐8, and COX‐2 induced inflammatory response	[41]
*Bacteroides fragilis*	Production of toxins, promotion of breakdown of E‐cadherin	[42]
*H. pylori*	Production of VacA	[43]

Microbiota and mechanism of carcinogenesis of gall bladder cancer		
*H. pylori*	Inflammation‐induced tumorigenesis by inducing release of various pro‐inflammatory mediators like TNF‐α, IL‐1, IL‐6, and other vasoactive substances, and aggravation of mucosal lesions	[44, 45]
*Salmonella typhi*	Result in a chronic inflammatory state and subsequent development of cancer. The typhoid toxin is carcinogenic and causes alterations in the cell cycle and DNA damage	[45, 46, 47, 48]
*Clostridium*, *Bifidobacterium*, *Peptostreptococcus*, *Bacteroides*	Have ability to form a biofilm, thereby resisting cellular and DNA damage caused by bile.	[45, 46, 47]
*Collibacillus*, *B. fragilis* , *Klebsiella*, *C. perfringens,* and *Clostridium* spp.	Interfering with the enterohepatic circulation	[49]
*E. coli* , *E. faecalis* , *Klebsiella, Enterobacter*	Both aerobic and anaerobic gram‐negative bacteria were found and may have a role in GBC	

Microbiota and mechanism of carcinogenesis of lung carcinoma		
*Bacillus* sp.	Production of toxins	[50]
*Mycoplasma* sp.	Production of ROS leading to DNA damage	[50]

Microbiota and mechanism of carcinogenesis of breast cancer		
Proteobacteria, Firmicutes, Actinobacteria, Bacteroidetes and Verrucomicrobia	Pyrosequencing V4 16S rDNA	[51]

Abbreviations: COX‐2, cyclooxygenase‐2; E‐cadherin, epithelial cadherin; IL‐1, interleukin‐1; IL‐8, interleukin‐8; NF‐κB, nuclear factor kappa‐light‐chain‐enhancer of activated B cells; ROS, reactive oxygen species; VacA, vacuolating cytotoxin A.

**FIGURE 1:**
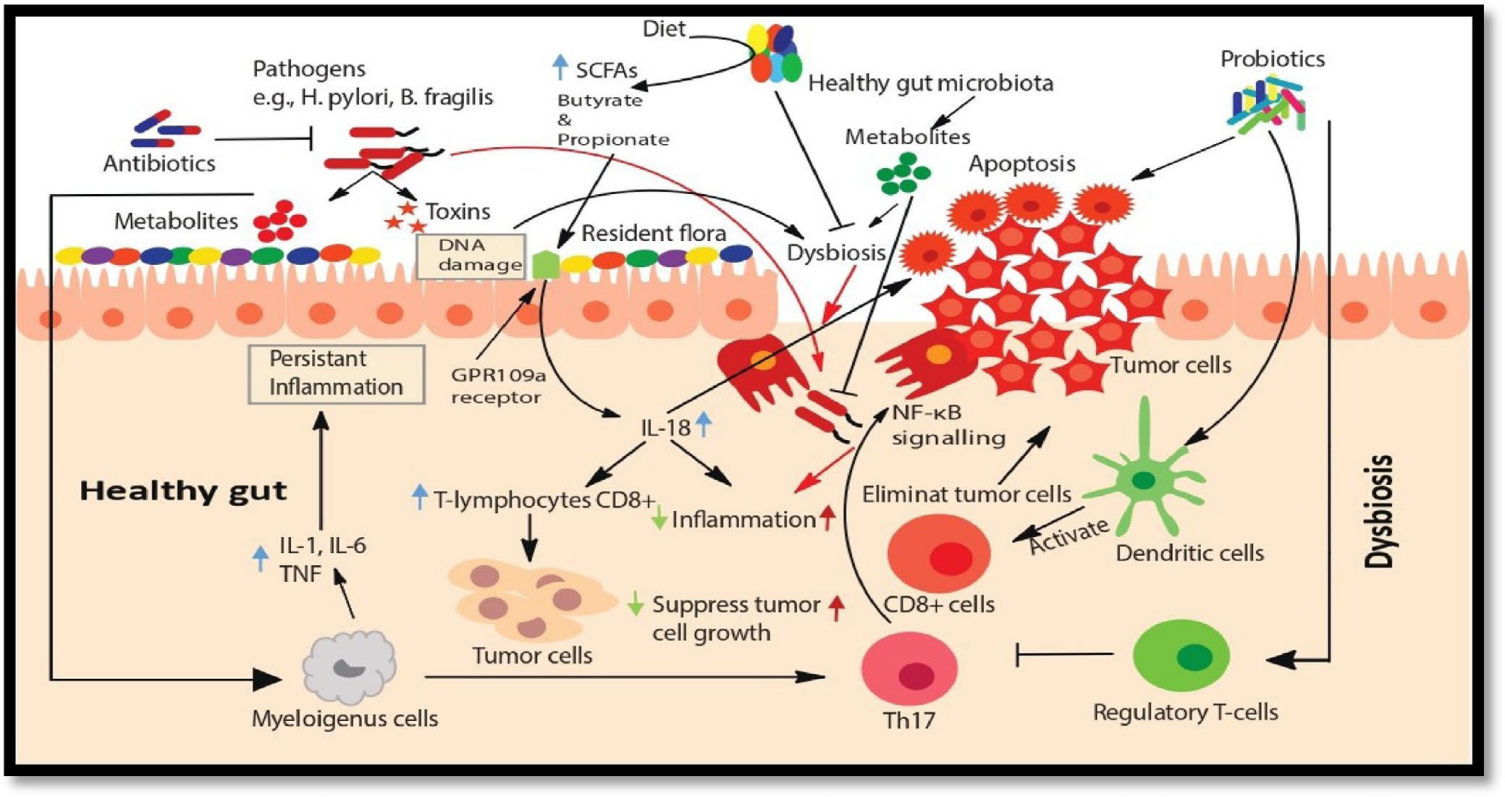
Interactions between the gut microbiota and cancer development. *Source:* Akbar et al. [53].

The improvement of intestinal barrier, bacterial translocation, and preservation of the gut microbiota's homeostasis and composition are all favored by the amelioration of microbiota brought about by probiotic consumption; healthy microbiota also demonstrates detoxifying activity against chemical genotoxic agents. Dysbiosis, disruption of the homeostasis of microbiota can make microbiota a carcinogenic agent. As indicated in Figure 1, through a variety of mechanisms, such as the production of microbial‐derived metabolites, bacterial‐derived genotoxins, host defense and inflammatory pathway modulation, oxidative stress induction, anti‐oxidative defense imbalance, and tumor microenvironment modulation, certain gut MOs may actually be involved in progenotoxic and pro‐carcinogenic processes [54].

The first pathogenic bacterium identified to have a role in the pathogenesis of cancer was *Helicobacter pylori* , categorized as a class I carcinogen by the World Health Organization (WHO). The bacteria can produce the virulence factor CagA (the product of the cytotoxin‐associated gene A), which provokes degradation of the p53 tumor suppressor gene in gastric epithelial cells, and hence enhances the increase in gastric cancer [55]. In addition, the *H. pylori* infection results in chronic inflammation, which in turn produces gastritis and, in some cases, stomach cancer. Other investigations demonstrated that *H. pylori* stimulate the β‐catenin signaling pathway, which in turn promotes carcinogenesis. In contrast, *H. pylori* 's function in the early stages of gastric carcinogenesis is supported by the fact that its eradication lowers the risk of gastric cancer in those who are infected [56].

Through interfering with the immune system, microbiota play a major role in both the development of disease and the preservation of host physiological functioning. Dysbiosis in microbiota, when disturbed, can result in the development of cancer. Several studies have shown that, even in the absence of a microbiome, exposure to Membrane‐acting Antimicrobial Peptides (MAMPS) and other metabolic products generated by bacteria can cause cancer in a number of human organs. Through anatomical connections, these metabolites are transported from the gut to the designated organs [1, 57]. SCFAs, which are produced when dietary substrates are altered by intestinal MOs, are important for inducing apoptosis in cancer cells, regulating the expression of tumor suppressor genes by inhibiting HDACs, and controlling cellular glucose metabolism [58].

The interaction of gut microbiota and cancer could be via various ways including contact‐dependent interactions that happen locally at the mucosal surface or within primary lymphoid organs (bone marrow and the thymus), and secondary lymphoid organs (lymph nodes and the spleen or the tumor microenvironment). The other way is via contact‐independent interactions that occur systematically through microbial metabolites and outer membrane vesicles in circulation. In a specific manner, gut microbes interact directly with the mucosal surface of the GI tract, finally resulting in genotoxic effects, epithelial cell proliferation, loss of cellular polarity, intestinal metaplasia. The hematopoiesis of the thymic and bone marrow could be stimulated by microbiota as shown in Table 1 that summarizes the association of bacteria with carcinogenesis [38].

For nearly a century, the identification of MOs in tumor tissues has been carried out, and there are identified characteristics of cancer in the past two decades. The ability to sustain proliferative signals, avoid growth suppressors, resist cell death, have infinite replicative potential, induce angiogenesis, activate invasion and metastasis, the capacity to avoid immunological destruction and the ability to rewire energy metabolism are the six fundamental hallmarks [59].

Studies have investigated the connection between cancer and microbiota in a number of solid tumors. Nejman et al. [60] discovered that bacteria can live in tumor tissues' macrophages and epithelial cells by analyzing the microbiota composition of over 1500 tumor samples. Tumor sample cultures can be used to cultivate living MOs. Numerous techniques, such as lipopolysaccharide (LPS) and lipoteichoic acid immunohistochemistry, as well as 16s ribosomal RNA (16S‐rRNA) sequencing, are used to investigate the bacterial makeup in different forms of cancer. The investigation of the intra‐tumoral microbiome resulted from the advancement of third‐generation sequencing tools. DNA sequencing of the bacterial 16S‐rRNA gene is an effective method for determining the makeup of the bacteria in cancer tissues [61].

## 3 Gut Microbial Modulation in Anticancer Therapies

The gut microbiota modulates pathogenesis of cancer because of its potential to synthesize various antitumor compounds, besides regulating immunity and host inflammatory cascades. These aggregate mechanisms may explain the strong impact of microbiota in the efficacy of different cancer therapies [55]. Antibiotics are compounds that destroy all populations of bacteria without discriminating between beneficial and dangerous strains. They cause gut dysbiosis which is characterized by a reduction in the abundance of the commensal community and a loss of richness of the microbial ecosystem. The emergence of opportunistic infections is encouraged by this, and host‐microbial interaction and microbiota function are negatively impacted. Antibiotic treatment is a hot topic of conversation among cancer patients since it is frequently given in combination with chemotherapy and cancer surgery. Antibiotics have been shown to accelerate the progression of several malignancies, including breast and melanoma. Nevertheless, they were helpful in cases of pancreatic cancer and other tumor types [51].

Chemotherapeutic medicines are metabolized by the gut flora; these gut microbiotas can either activate or deactivate the medication. The degree of a drug's effectiveness can be determined by the presence of a certain type of gut flora. It has been established that the intestinal microbiota affects the effectiveness or toxicity of several chemotherapy medications, including irinotecan, platinum salts, and cyclophosphamide (CTX). It has also recently been demonstrated that the composition of the gut microbiota plays a significant role in the mechanisms of action of immunogenic chemotherapy and the efficiency of anticancer immune responses [37].

**FIGURE 2:**
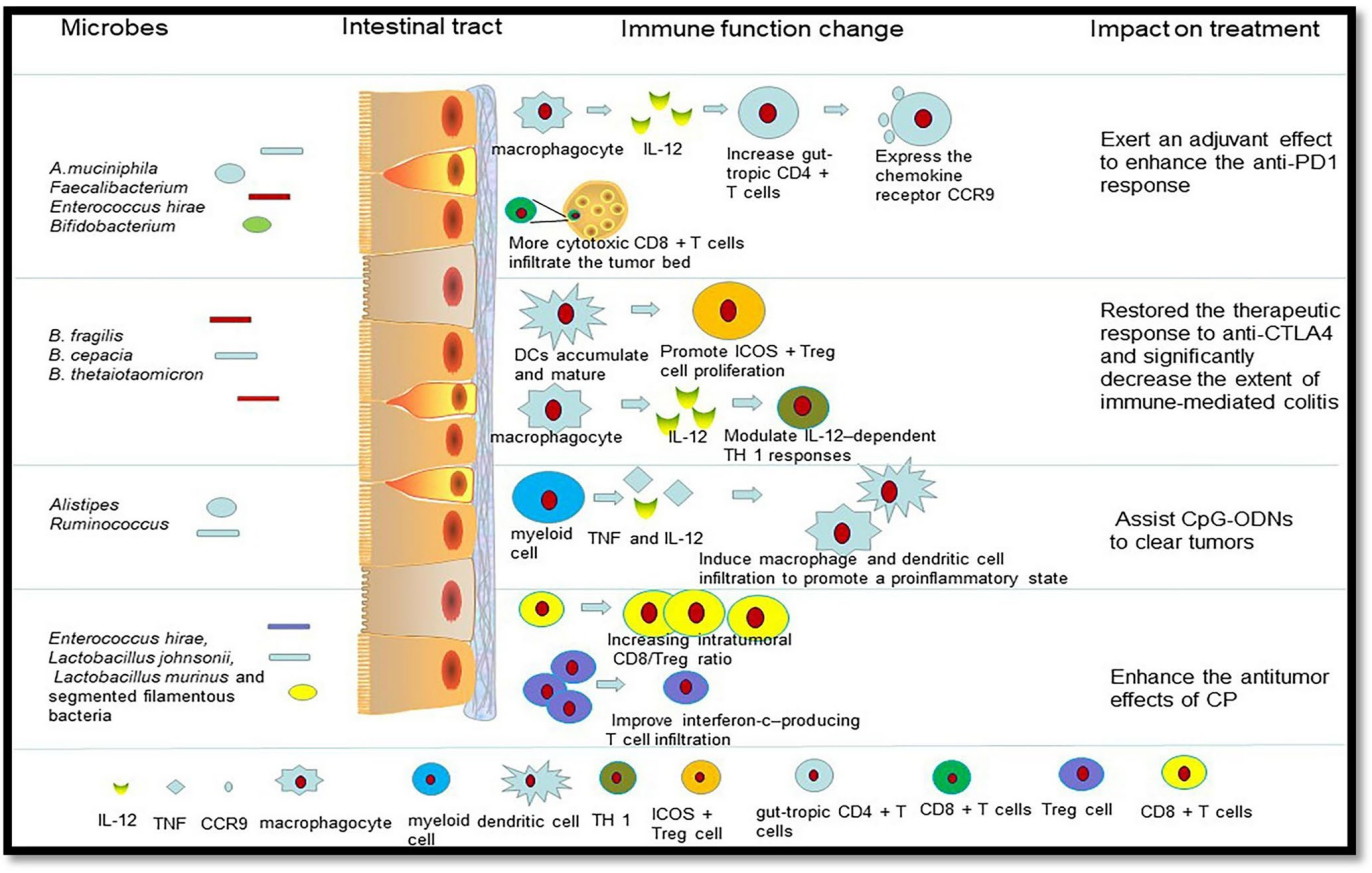
The microbiota influences drug toxicity and efficacy. *Source:* Ma et al. [62].

Changes in the gut microbiota are linked to resistance to immune checkpoint inhibitors (ICIs) or chemotherapy treatments, whereas adding other bacterial species to the diet helps the body respond to anticancer medications as shown in Figure 2. Growing data suggest that altering the gut flora may improve the effectiveness of anticancer medications [62].

The use of ICIs marked a paradigm shift in the treatment of cancer as monotherapy or in combination. Although they have produced unprecedented outcomes and are currently the standard of care for several solid tumors, a substantial proportion of patients obtain limited benefits, and efficacy that can vary even among patients with the same cancer type and comparable biomarkers. Following the emergence of the gut microbiome as a key regulator of host immunity, accumulating evidence suggests that the microbiome may serve as both a predictive biomarker of ICI response and as a therapeutic target to enhance efficacy. As a result, the inclusion of microbiota‐based approaches into ICI therapy becomes a promising strategy to improve clinical outcomes [63, 64].

Other recent investigations have demonstrated that gut microbiota combined with ICIs synergistically promotes the expansion of CD8^+^, CD4^+^, and γδ T cells and reversed CD8^+^ T cell exhaustion by fostering their differentiation into memory or effector phenotypes, thereby amplifying the antitumor immune response. Tregs are a specific type of immunosuppressive T cells characterized by the expression of CD4^+^, CD25^+^, FOXP3^+^ which directly suppress T cell proliferation and activation through cell‐to‐cell contact and secreting inhibitory cytokines with potent immunosuppressive properties, such as IL‐10 and TGF‐β. Furthermore, the gut microbiota has been reported to synthesize and transform a multitude of metabolites that can enhance the efficacy of ICIs by modulating the local and systemic antitumor immune responses [64].

Evidence associates specific microbial features, taxa, and bioactive metabolites with enhanced antitumor immunity, while disruptions with antibiotic exposure are linked with reduced outcomes. Translational studies demonstrate that microbiome‐based intervention, including FMT, biotics supplementation, and engineered microbial strains, can enhance ICI efficacy or mitigate immune‐related toxicities. Despite encouraging early clinical signals, broader implementation requires methodological rigor, standardized protocols, and innovative trial designs that account for host and environmental factors. For clinicians, the most immediate strategies involve prudent antibiotic stewardship and patient enrollment in microbiome‐focused clinical trials. Overall, the gut microbiome is a promising biomarker and a therapeutic target, representing a new frontier for personalizing immunotherapy and improving patient outcomes in oncology [63].

The normal and pathological immune responses for cancer therapies are shaped in part by gut bacteria. Microbial populations and mucosal immunity of a host have coevolved, and several strategies have been devised to preserve homeostasis. Nevertheless, the immune system reacts to harmful MOs when they upset this delicately balanced ecology. It may also alter the immune response to malignancies and the tumor microenvironment [62].

Numerous lines of evidence suggest that the way anticancer therapy works on individual patients varies because of various host genes, various tumor mutations, various environmental variables, and, to a lesser extent, variations in the composition of the gut microbiota. As a result, altering gut microbiota may be a useful strategy for raising the effectiveness of conventional anticancer treatment. Microbiota‐targeted combination therapy aims to reduce the number of genera that may compromise the effectiveness of anticancer therapy, for example by targeting the strains that may induce resistance with antibiotics. However, since dysbiosis may result from anticancer therapy, maintaining the beneficial bacterial strains of patients can also help to effectively restore the microbiota community and their capacity to interact with drugs [65].

Currently assessing the diversity of gut microbiome becomes an essential component in understanding its role in modulating anti‐cancer therapeutic responses and in the design of microbiome‐informed clinical studies. Alpha diversity microbial richness and evenness within a sample is widely used as a surrogate marker of the stability of the gut microbial ecosystem. The reduced diversity is often associated with dysbiosis and reduced responses to immunotherapy. In contrast, Beta diversity quantifies the differences in microbial composition between individuals or groups and is particularly important for identifying microbiome signatures that are linked with therapeutic response, resistance, or disease progression [66].

For clinical trial use, alpha diversity could be used as a biomarker to monitor the effects of treatment on the stability of the microbial community, whereas beta diversity can be used to stratify patient populations. Collectively, these measures offer a useful strategy for patient stratification and discovery of biomarkers besides incorporating microbiome studies into precision oncology trials. Therefore, incorporating the two measures of diversity into microbiome studies offers insight into host‐microbiome dynamics and helps pave the way for the development of predictive biomarkers and therapeutic treatments for cancer patients [67].

### 3.1 Modulating Gut Microbial Composition in Preventing Associated Toxicity and Side Effects From Anticancer Therapy

The gut microbiota promotes the efficacy of anti‐programmed cell death protein 1 (PD)‐L1 through activation of the dendritic cells (DCs), which there by follow to boost CD8‐positive T cell responses to rise gut tropic CD4^+^ T‐ cells to defeat tumors. Moreover, the gut microbiota enhances maturation of DC and regulate IL‐12‐dependent TH 1 responses so as to restore the therapeutic response towards anti‐CTLA4. The side effects of immunotherapy have also been associated with the function of microbiota. When germ‐free mice receiving anti‐CTLA‐4 monoclonal antibodies were monopolized with *B. fragilis* , plasmacytoid DCs accumulated and matured in mesenteric lymph nodes. DCs can promote ICOS+ Treg cells to proliferate in the lamina propria. The gut microbiota helps CpG‐ODNs to promote myeloid cells to secrete proinflammatory cytokines such as TNF and IL‐12, which then activate macrophage and DC infiltration to promote a proinflammatory state. The body develops an antigen‐specific adaptive T‐cell antitumor immunity to clear tumors in the proinflammatory microenvironment. Some intestinal microbiota affects antitumor efficacy of CP. *E. hirae* translocation, that could improve the intratumoral CD8/Treg ratio. The gram‐negative *Barnesiella intestinihominis* was also found to improve interferon‐c‐producing T cell infiltration in cancer lesions to enhance the antitumor effects of CP [62].

Changing the gut flora may have a significant impact on how well anti‐cancer treatments work. Table 3 summarizes major microbial interventions and effects on anticancer treatments. In actuality, patients' microbiomes can be altered by immunotherapy, chemotherapy, and radiation therapy; but patients' reactions to these treatments can also be significantly impacted by the makeup of their microbiomes. In order to ultimately improve patients' therapeutic outcomes, it is crucial to determine which factors have the ability to affect the gut microbiota and, consequently, to develop novel techniques to manage the gut microbiome. In particular, microbiome‐related therapies may be essential to reduce toxicity associated with anti‐cancer therapy and enhance its effectiveness [76].

**TABLE 3:** Clinical trials on gut microbial modulation to prevent anticancer therapy‐related toxicity and side effects.

Microbial interventions on different cancer types	Primary outcome measures	References
FMT via colonoscopy on Malignant genitourinary system neoplasm	FMT associated adverse events. Clinical response/remission of immune related diarrhea/colitis	[68]
FMT capsules on renal cell carcinoma	Grade 3 or higher immune associated colitis from the start of treatment with ipilimumab and nivolumab to 120 days after treatment	[69]
Probiotic on pelvic cancer	Grade 3 enteritis	[69]
Dietary intervention: a high‐fiber diet rich in polyunsaturated fatty acid on CRC	Rate of anastomotic leakage surgical site infection	[70]
Probiotic capsules with *Bifidobacterium longum* , *Lactobacillus acidophilus* and *Enterococcus faecalis* on breast cancer	Cancer related cognitive impairment	[71]
Probiotic on CRC	Prevention of grade 3–4 diarrhea by probiotics in patients treated by irinotecan‐based chemotherapy	[72]
Probiotic for prostate cancer; gynecologic cancers	The efficacy of probiotic	[73]
FMT for renal cell cancer	Rate of patients who experience resolution of diarrhea 4 weeks after the end of treatment	[74]
Rectal cancer Dietary supplement: Oat bran and blueberry husks on rectal cancer	Action of synbiotics on irradiated GI mucosa in rectal cancer treatment	[75]
Dietary supplement: Inulin, fructo‐oligosaccharide and maltodextrine on endometrial neoplasm	Alterations in *Lactobacillus* and *Bifidobacterium* populations	[69]

## 4 Current Implications and Future Perspectives of Gut Microbiota in Cancer Therapy

Investigations revealed that gut microbiota affects the efficacy and toxicity of cancer therapies including chemotherapeutic agents, radiation therapy, and immunotherapy. The resulting alterations in microbiota composition are influenced by patient demographics, physiological and nutritional status, use of polypharmacy, and cancer stage [77].

Probiotics, the live Mos, have multiple health benefits when used in sufficient amounts. They change the microbial composition by replacing pathogenic microbes with the beneficial one [78]. Regulation of the immune system, reduction in colitis and blood cholesterol, and inhibition of pathogenic bacteria are among their health benefits [79]. Various investigations showed that probiotics can prevent aggregation of pathogenic microbes by competing for nutrients or adhering to epithelial cells or mucus and, therefore, aid in preventing intestinal infections. Furthermore, they can also produce metabolites that inhibit the growth of pathogens [80].

Prebiotics, the dietary or food components, provide health benefits by keeping gut microbiota healthy. Clinical trials have shown that the use of prebiotics enhances the abundance of probiotic strains, like *Ruminococcus*, *Faecalibacterium*, *Roseburia*, and *Akkermansia* [81, 82]. Oligosaccharide‐prebiotics inhibit the aggregation of pathogens because of their interaction with bacterial receptors and blocking the attachment of pathogens to epithelial cells [83]. Investiagation on polydextrose showed its beneficial effects on keeping healthy gut microbiota. Similarly, fructans and galacto‐oligosaccharides raise the abundance of beneficial bacteria, like *Bifidobacterium* and *Lactobacillus*, and increase fecal butyrate concentration [84]. Inulin, a polysaccharide from artichokes, bananas, asparagus, and wheat, reduces the formation of precancerous lesions via inhibiting the activity of glucuronidase and decreasing pH and concentration of indole, phenol, and p‐cresol in the colon. Additionally, its use has also been shown to increase the availability of *Bifidobacterium*. Agro‐oligosaccharides can change the formation of SCFAs and secondary bile acids. Polysaccharides from *Lachnum* sp. also bring about gut microbiota alterations and are implicated in reducing inflammation and tumor incidence [69, 70, 85].

### 4.1 Chemotherapy

Studies revealed that gut microbiota affects the activity and efficacy of various cancer treatment options [71, 80]. The modulation of the host responses might be by mechanisms targeting translocation, immunomodulation, metabolism, enzymatic degradation, and reduced diversity and ecological variation [72, 73].

CTX can alter the gut microbiota and enhance translocation of G^+ve^ bacteria into secondary lymphoid organs, resulting in the generation of T helper (Th17) cells. A study on a mouse model where G^+ve^ bacteria were killed by antibiotics showed resistance to the anti‐tumor effects of CTX [74, 75], while regaining the microbiota ameliorates the efficiency of CTX. Other mouse model studies have also shown that CTX's antitumor activity had increased in the presence of bacteria, such as *Enterococcus hirae* , *Lactobacillus johnsonii* , and *Barnesiella intestinihominis* [86].

Tumor retardation potential of another chemotherapeutic agent, oxaliplatin, is dependent on microbiota. Its efficacy was reduced because of decreased intratumoral ROS generation in germ‐free mice. In addition, when patients were treated with antibiotics, the recruitment of immune cells that mediates tumor regression was decreased, and their proinflammatory effect was also reduced [49].

### 4.2 Immunotherapy

Immunotherapy has also been employed in treating different cancers. ICIs are widely used in immunotherapy that activate T cells so as to initiate anti‐tumor responses. ICIs are usually monoclonal antibodies that prevent PD‐1 from interacting with its ligand, PD‐L1, or enable cytotoxic lymphocyte‐mediated attack on tumor cells by targeting cytotoxic T‐lymphocyte antigen 4 (CTLA‐4) [87, 88]. Investigations revealed that gut microbiota can regulate immunotherapy, and various findings showed that certain bacteria including *Bifidobacterium* [89], *Faecalibacterium* [90, 91], and *Akkermansia* [91] are positively associated with immunotherapeutic response. Besides, Tanoue et al. [92] identified 11 bacterial strains that promote the therapeutic efficacy of ICI. The gut microbiota is also related to ICI induced colitis [93].

Immunotherapy induced colitis in patients treated with antibiotics was significantly reduced after treatment with intestinal microbiota transplantation (IMT) using fecal slurry from healthy donors [77, 94]. Similarly, IMT from patients responding to anti PD L1 to germ‐free mice or antibiotic‐treated mice improved the efficacy of immunotherapy [91]. The use of probiotics including Bacteriodales and Burkholderiales, and FMT, improves ICI induced colitis [94].

In the process of tumor formation, tumor cells frequently utilize different mechanisms to influence themselves and nearby cells which provide a favorable environment for the proliferation and metastasis of tumor cells. Studies have indicated that tumor cells increase the concentration of lactate at the tumor site by the use of glycolysis, and the buildup of lactate is frequently associated with a reduction in the immune response against the tumor within the TME [24].

Tumor cells regulate a complex and evolving TME by secreting various factors that influence the surrounding stroma. The components of TME include adaptive immune cells: T cells and B cells, and innate immune cells: DCs, macrophages, neutrophils, myeloid‐derived suppressor cells (MDSCs), innate lymphoid cells (ILCs), and natural killer (NK) cells [95].

The anti‐tumor immune response of the body depends highly on adaptive immune cells, particularly T cells. In the absence of CD8+ T cells, there will be impairment of the immune response within the TME. Treg cells, a subset of CD4+ T cells identified by the expression of the transcription factor FOXP3, strongly suppress anti‐tumor immune responses. The other elements in the adaptive immune cells' population in TME are B cells, which can produce cytokines that directly act on T cells to modulate anti‐tumor immune responses. Innate immune cells also contribute to the anti‐tumor immune response: DCs, neutrophils and their subset MDSCs, and ILCs support cross‐priming of tumor‐specific T cells; macrophages engulf pathogens and components of the TME; and NK cells provide direct cytotoxic activity to help initiate and sustain the immune response [5, 96].

Immune cells are highly important to maintain a healthy microbial community, and their function is modulated by microbiota and its metabolites. For instance, the Th17/Treg balance is pivotal in cancer progression, and excessive inflammation because of Th17 cells or excessive immune suppression induced by Treg cells may lead to carcinogenesis. Metabolites of microbiota: SCFAs, MTCs, and secondary BAs can increase Th17 cell populations while reducing Treg cells, thereby contributing to anti‐tumor effects; in contrast, microbiota‐derived hydrogen sulfide can disrupt this balance and promote tumor progression [5].

### 4.3 Fecal Microbiota Transplantation

FMT, transferring microbials from a donor to a recipient, is a recent biotherapeutic approach to restore the normal gut microbial ecosystem [97]. A study by Rosshart et al. [97] showed FMT from wild mice transferred to laboratory mice provide resistance against DSS/AOM induced colorectal tumorigenesis. Their method of acquiree, safety issues, and potentially limited efficacy of FMT, however, have prevented its wider use. With this regard, patients that took FMT were reported adverse effects like diarrhea, constipation, and abdominal distension [98]. Another possible risk with FMT is the transmission of multi drug resistant bacteria possibly leading to a life‐threatening infection such as *Escherichia coli* bacteremia. The transmission of microbiome related chronic diseases, such as gastrointestinal, cardiometabolic, and autoimmune disorders are also risks associated with FMT. Various Studies reported that transferring human feces from obese individuals to germ free mice had induced obesity and other study also reported the transmission of atherosclerosis after FMT [80, 99, 100, 101].

Studies have shown the link between dysbiosis and cancer because of the presence of microbes in the digestive tract and tumor tissue. It has been shown to have significant influence on carcinogenesis, tumor progression, and therapeutic response in both humans and experimental animals. Our knowledge of the systemic impacts is still far more limited, despite the fact that many of the mechanisms behind the local effects have been described recently. Comprehensive comprehension of these pathways in humans and experimental animals can help us target them therapeutically, which could lead to significant advancements in the prevention and treatment of cancer [76, 102]. Because xenobiotics influence both microbiota composition and their functional capacity, the upcoming drug trials should therefore consider the interplay of genetic, environmental, and microbiome factors [77].

The microbiome has the potential to be a significant biomarker or interventional target for bettering cancer treatments in the future, marking a hopeful advancement towards precision and personalized medicine. Numerous observational studies have shown correlations between the composition of the gut microbiota and the effectiveness of anticancer medications. However, more research using animal models or large‐cohort clinical trials is needed to determine the causal involvement of microbiota in therapeutic efficacy and toxicity. Furthermore, when extrapolating findings from animal models to humans, consideration should be given to the ways in which humans and animal models interact with microbiota and medications [103].

The homogeneity and consistency of the mechanistic understanding of the microbial effect on cancer could not currently be guaranteed due to lack of uniform methodology, which includes variations in sample selection and collection, technology, data quality, and resource analysis. Results from the same people could vary greatly in different samples. For example, the microbiota colonized on the mucosa of the digestive system and those in feces have similar, but different, compositions and levels of richness [38, 104].

Distinct microbiota stratification will need to be prioritized in the near future. A single microbe profile, which is similar to a single‐cell sequence and can help identify exact influencing pathways, may be used to study the unique strain found in various hosts. Additionally, certain specialized preclinical models, such as organotypic tumor spheroids produced from patients in short‐term 3D cultures in the same environmental conditions, can be utilized to validate the results in vitro and connect the molecular pathways to practical uses [38, 105].

The emergence of potent computational tools and advancements in sequencing technology have caused a paradigm change in research from association‐based to mechanism‐based approaches. The presence or lack of specific bacterial species alone doesn't tell us much about how the gut microbiota contributes to cancer. In fact, a great deal of research has been done to determine the underlying mechanisms and causal relationships between bacteria and malignancies. Furthermore, changed fungal microbiota and viromes have been linked to CRC, indicating that these microbes may interact with gut bacteria to affect how patients react to cancer treatment. Improving the prognosis of cancer patients requires an integrated examination of the gut microbiota and its interactions with the host, anti‐cancer medications, and other external variables [62].

The gut microbiota has a major role in the therapeutic efficacy and efficiency of anticancer therapies. But it affects both intestinal and extraintestinal tissues' reaction to anticancer treatment. The study of the molecular and cellular pathways governed by host‐microbiota interaction is hampered by complex biological systems. However, in order to produce systemic clinical trials that might offer fresh and improved insights into the underlying mechanisms of these pathways, interdisciplinary approaches must be used [1].

The link between microbial species, their metabolites, and immune cells is a promising area that requires continued investigations to fully exploit the association of gut microbiota and immunotherapy. To create more precise personalized medicine, it is crucial to scrutinize how various tumors are influenced by gut bacteria [105].

## 5 Conclusion

Numerous preclinical and clinical experiments have explored gut microbiota as a new research avenue, with encouraging outcomes in enhancing treatment efficacy and mitigating side effects related to treatment. To date, there are various strategies ranging from FMT to oral probiotic feeding to alter the gut microbiota's composition, many of which have already received approval for standardized clinical use. Though different bacterial species can cause different clinical outcomes, more research is still required to identify more precise and profound underlying mechanisms in the relationship between gut microbiota and anticancer treatment.

The fact that different subtypes of the same bacterial strain can elicit different host immune responses is pivotal to identifying the variability in host immune responses. Interestingly, research is presently underway to determine how gut microbiota mapping could be utilized as a predictive biomarker to gauge patient clinical results. This could lead to improved patient outcomes overall and more focused clinical applications down the road. Finally, even though there are still a lot of obstacles to overcome, the importance of gut microbiota and its potential for developing novel anti‐cancer treatments cannot be emphasized, and a comprehensive strategy that integrates microbial modulation therapy into the current cancer management system needs to be investigated. Ongoing investigations into the roles of specific microbial species and their metabolites could offer opportunities for the development of novel diagnostic biomarkers and therapeutic options with a focus on individual microbial profiles. Therefore, complex interactions need rigorous exploration with future studies to optimize microbiota‐based therapies for cancer.

## Author Contributions

**Awgichew Shewasinad Yehualashet:** conceptualization, investigation, resources, writing – original draft, writing – review and editing. **Eleni Teklu Fersha:** conceptualization, investigation, writing – review and editing, resources, writing – original draft. **Berhan Begashaw Yikna:** writing – review and editing, resources, investigation. **Kassahun Dires Ayenew:** investigation, writing – review and editing, resources.

## Funding

The authors have nothing to report.

## Consent

The authors have nothing to report.

## Conflicts of Interest

The authors declare no conflicts of interest.

## Data Availability

The authors have nothing to report.
